# MR imaging of the airways

**DOI:** 10.1259/bjr.20220630

**Published:** 2023-02-21

**Authors:** Juergen Biederer

**Affiliations:** 1 Christian-Albrechts-Universität zu Kiel, Faculty of Medicine, Kiel, Germany; 2 University of Latvia, Faculty of Medicine, Raina bulvaris, Riga, Latvia; 3 Translational Lung Research Center Heidelberg (TLRC), Member of the German Lung Research Center (DZL), Im Neuenheimer Feld, Heidelberg, Germany; 4 Department of Diagnostic and interventional Radiology, University Hospital of Heidelberg, Heidelberg, Germany

## Abstract

The need for airway imaging is defined by the limited sensitivity of common clinical tests like spirometry, lung diffusion (DLCO) and blood gas analysis to early changes of peripheral airways and to inhomogeneous regional distribution of lung function deficits. Therefore, X-ray and computed tomography (CT) are frequently used to complement the standard tests.

As an alternative, magnetic resonance imaging (MRI) offers radiation-free lung imaging, but at lower spatial resolution. Non-contrast enhanced MRI shows healthy airways down to the first subsegmental level/4^th^ order (CT: eighth). Bronchiectasis can be identified by wall thickening and fluid accumulation. Smaller airways become visible, when altered by peribronchiolar inflammation or mucus retention (tree-in-bud sign).

The strength of MRI is functional imaging. Dynamic, time-resolved MRI directly visualizes expiratory airway collapse down to the lobar level (CT: segmental level). Obstruction of even smaller airways becomes visible as air trapping on the expiratory scans. MRI with hyperpolarized noble gases (^3^He, ^129^Xe) directly shows the large airways and peripheral lung ventilation. Dynamic contrast-enhanced MRI (DCE MRI) indirectly shows airway dysfunction as perfusion deficits resulting from hypoxic vasoconstriction of the dependent lung volumes. Further promising scientific approaches such as non-contrast enhanced, ventilation-/perfusion-weighted MRI from periodic signal changes of respiration and blood flow are in development.

In summary, MRI of the lungs and airways excels with its unique combination of morphologic and functional imaging capacities for research (*e.g.,* in chronic obstructive lung disease or asthma) as well as for clinical imaging (*e.g.,* in cystic fibrosis).

## Introduction

Scientific research and advanced concepts for clinical monitoring of airway disease have raised the need for reliable biomarkers. These would be expected to reflect the status of the airway system at a given time point and should allow for follow-up and monitoring disease activity. In basic clinical practice, this is done with pulmonary function tests (PFT, spirometry), lung diffusion tests (DLCO) and blood gas analysis.

The potential role of imaging becomes evident against the background that standard global pulmonary function tests (global volumetry, pneumo-tachymetry, FEV1 etc.) per definition do not account for regional changes of lung function. Focal disease with regional functional impairment can be compensated by healthy or recovering portions of the lung at other locations. Therefore global tests can be insensitive to early disease in the lung periphery or dynamic disease development with changing distribution of pathology all over the airways and lungs.^1^

Many clinical tests such a spirometry measure global changes of airway resistance. From the trachea, airway resistance initially increases towards its maximum around the fourth-eighth airway generation (subsegmental level). From there, it decreases again as a consequence of further branching of the airways and continuous increase of total cross-sectional area reaching a minimum at the 15^th^ generation and beyond.^2^ Therefore, any measurement of total airway resistance mainly reflects changes in the segmental and subsegmental airways.^3^ Consequently, standard pulmonary function tests are highly efficient to measure changes of the central airways related to obstruction or bronchospasms (asthma, bronchospasms in airway-type COPD) but they hardly measure dysfunction in small airways which are <2 mm in diameter.^4^ Any disease activity in the lung periphery is therefore underrepresented in PFT unless it becomes significant.

Non-standard clinical tests to measure small airways dysfunction beyond standard spirometry/FEV1 are the multiple breath nitrogen washout test (MBNW) and the forced oscillation technique (FOT). MBNW uses the washout-kinetics of nitrogen as inert tracer gas from the lungs during tidal breathing and allows for calculation of the functional residual capacity (FRC) and the lung clearance index (LCI). FOT is based on the response of the respiratory system to small externally produced oscillatory (vibratory) forces applied with a loudspeaker from the mouth. Responses to higher frequencies correspond to the mechanical properties of the large airways, lower frequencies to the small airways, making the test particularly sensitive to small airways obstruction. Both tests allow for the detection of global ventilation heterogeneity due to small airways dysfunction. This is considered an important marker of asthma disease activity, even in the absence of abnormalities in standard spirometry.^3–6^ Finally, lung diffusion capacity tests (DLCO) give insight into the overall impairment of lung function. All these tests assess global airway function, but do not allow to draw conclusions on the regional distribution of lung pathology.

From imaging, it is expected to contribute information beyond the scope of these tests.^7^ The most frequently applied imaging technique to complement clinical PFT with airway imaging is computed tomography (CT). However, CT *per se* is not available without radiation exposure, which limits its frequent and repeated use, in particular in children. Among all alternative technologies, MRI of the lung comprises morphological and functional aspects more than any other imaging modality.^1^ It is therefore the aim of this article to highlight and summarize the available technology and diagnostic scope of lung MRI for airway imaging.

## MR-imaging of the tracheobronchial morphology

MRI with practical “easy-to-use” protocols for common clinical indications is being increasingly appreciated as an alternative to CT. Two- and three-dimensional (2D and 3D) imaging without radiation exposure as well as superior soft-tissue contrast and exclusive morpho-functional imaging capacities beyond the scope of CT are offered. Fast imaging protocols are based on breath hold-acquisitions or triggering/gating to compensate for motion artefacts and comprise useful components for airway imaging.^8,9^

*T*_2_-weighted fast spin-echo sequences are typically acquired with or without fat-signal suppression and provide high contrast for fluid detection. This is useful for the assessment of bronchial wall thickening and mucus plugging. Bronchial wall imaging can be further improved with black-blood preparations using heavy T2-weighting (longer effective echo time).^10^ Black-blood preparation removes signal of pulmonary vessels, facilitating detection of bronchiectasis, mucus plugging and small nodules. Due to the long echo times, 3D-acquisitions are difficult to realize with *T*_2_-weighted images. Therefore, this part of the protocol is typically acquired in 2D-series.

Images with a mixed T2/*T*_1_-weighted signal are obtained with gradient echo steady-state free precession (SSFP) sequences, which enhance the visibility of material with water-like characteristics, such as mucus plugs in the airways. Two-dimensional SSFP sequences are very fast and can cover the entire thorax in a single breath-hold with a good image quality or be applied for cine-imaging at a single position, *e.g.,* for imaging respiratory dynamics and airway collapse. Due to the faster acquisition schemes, SSFP-sequences are available in 2D and 3D-mode.

*T*_1_-weighted images are typically acquired with three-dimensional gradient echo sequences using short and ultra-short echo times (3D-GRE). These are considered the most robust acquisition format for lung MRI and particularly useful to review central airway pathology. Gradient echo sequences with low flip angles produce proton-density weighted acquisitions which are frequently used to assess airways without the use of contrast agents and to detect air-trapping in end-expiratory images. Very fast *T*_1_-weighted 3D-GRE sequences are used to assess lung parenchymal perfusion when combined with contrast administration (dynamic contrast enhanced MRI/DCE-MRI, sometimes also referred to as“4D-MRI”).^8^

These morphologic MR sequences reach pixel-sizes down to 1.8 × 1.5 mm in breath-hold and 1.3 × 1 mm in triggered/gated acquisitions at slice thicknesses of 4–6 mm. Isotropic acquisitions in breath-hold may reach 2 × 2 x 2 mm. This allows for a visualization of bronchi down to the fourth generation (subsegmental level) in healthy subjects.^11–13^

Further increase of spatial resolution to voxel sizes below 1 × 1 x 1 mm and therefore approaching the scale of high-resolution CT can be achieved with self-gated UTE sequences.^14^ Ultra-short echotime or zero echotime imaging (UTE or ZTE) address the central challenges of pulmonary MRI: low proton density of the lungs and fast decay of the already low signal due to a very rapid transverse (R2*) relaxation rate. Images are typically acquired as a 3D volume over a couple of minutes, either as externally or self-navigated acquisition, which allows for retrospective reconstructions of different respiratory phases (4D-MRI).^15^ However, as with any gated approach, it remains difficult to achieve motion-free images over the whole volume, with the best results typically in the upper parts of the lung and in end-expiration.^15,16^ Compared to 3D-GRE imaging, UTE or ZTE imaging appears to provide a higher signal-to-noise ratio in the peripheral airways.^15^ In respect to field strength, 1.5 Tesla MRI remains superior to 3 T when using a 3D-UTE sequence offering better bronchi visualization, SNR and CNR, as well as less ringing artifacts.^17^

Being limited to visualization of airways in excess of 2–3 mm (fourth generation), MRI stays behind CT, where peripheral airways can be detected up to the eighth generation. This is still good enough to detect wall thickening of central airways using UTE MRI in asthma with comparable results to CT.^18^ The spatial resolution of UTE was found enough for the assessment of lobar bronchial metrics (bronchial wall area (WA), lumen area (LA) percentage of bronchial wall area (WA%) and plain wall thickness (WT)) to differentiate severe from non-severe asthma with the smallest measured bronchus having a luminal diameter of only 2.25 mm and good correlation with CT.^18^ However, due to a lower spatial resolution compared to HRCT and the susceptibility to motion artifacts, non-contrast-enhanced MRI appears to be not yet suitable as a screening test for subtle changes of airway structure. If the detection of such would be crucial for therapeutic decision, a HRCT for the baseline examination might still be advisory, to then switch to MRI for follow-up.^9^

**Figure 1.**
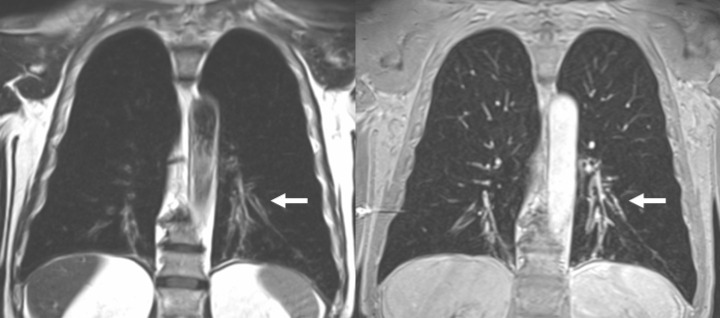
71-year-old male, COPD, bronchiectasis in both lower lung lobes. Images obtained in inspiratory breath-hold, *T*_2_-weighted fast spinecho (left) and 3D-gradient echo MR after iv. contrast application (right). MRI shows dilated airways with wall thickening and intense signal after iv. contrast application as sign of inflammation (arrows).

Much smaller airways up to the bronchiolar level (eighth generation and higher), become visible on MRI, when altered by peribronchiolar inflammation or mucus retention (tree-in-bud).^19^ Bronchiectasis can be easily identified by wall thickening and accumulation of fluid (Figure 1).^20^

**Figure 2.**
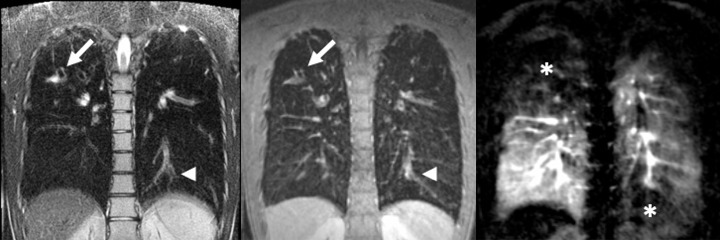
MRI of bronchiectasis and perfusion deficits in a 15-year-old patient with severe CF. *T*_2_-weighted fast spinecho (left), *T*_1_-weighted gradient echo (middle) and subtracted perfusion MRI (right) in coronal orientation show bronchiectasis (arrows), mucus retention (arrowheads) and resulting perfusion deficits from hypoventilation and hypoxic vasoconstriction (asterisks). *Images courtesy of M. Wielpuetz, Heidelberg, DE*.

Under the assumption that clinical pulmonary function tests are not sensitive enough to follow subclinical disease progression, imaging of airway involvement plays a central role in clinical monitoring and research of cystic fibrosis (CF) lung disease. MRI has been shown to be efficient in imaging bronchial wall thickening, bronchiectasis, mucus plugging, air-fluid levels, consolidation/infiltration, mosaic pattern air trapping and lobar or segmental destruction, with reasonable quality compared to CT.^21–25^ Based on a standardized MRI protocol,^8^ a semiquantitative scoring system for airway pathology^26^ and the validation of the concept in a multicenter setting,^27^ clinical imaging of airway disease with MRI has become state-of-the-art for imaging in cystic fibrosis (Figure 2).^1,9^

Similarly, MRI has been successfully applied for other diseases of the bronchial system related to muco-cilial dysfunction such asallergic bronchopulmonary aspergillosis (ABPA) or Kartagener syndrome.^28^ The sensitivity of the fast MRI protocol for key diagnostic features, *e.g*., of ABPA (bronchiectasis, consolidation, nodules, mucus retention) ranged from 68 to 100% with negative predictive values of 71 to 100%, the lowest for bronchiectasis and highest for mucus retention. Specificity and positive predictive values were excellent (100%).^28^ In research, morphologic MRI of the airways has become part of large multicenter-trials, *i.e*. for imaging of COPD.^29^

## Dynamic airway imaging

**Figure 3.**
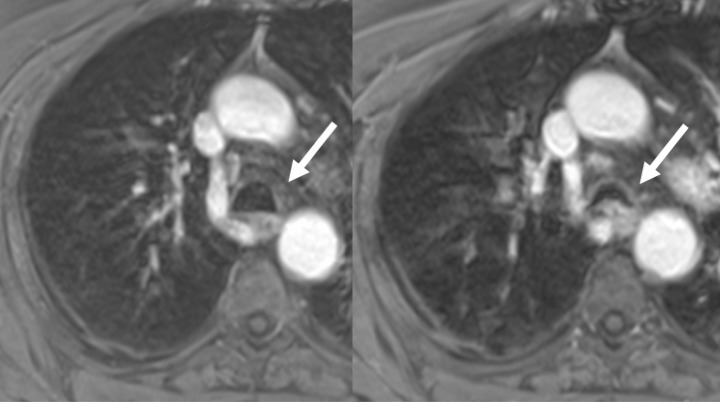
86-year-old male with COPD, expiratory collapse of the trachea in expiration. 3D-gradient echo MR scans in inspiratory (left) and expiratory breath-hold (right) obtained after iv. contrast application. In expiration, the tracheal lumen collapses by approximately 50% of the sagittal diameter (arrows)

Inspiratory and expiratory scans can be used to detect large airway collapse in expiration (Figure 3). Real-time dynamic imaging (cine-MRI) of the central airways over the whole respiratory cycle can be realized with fast 2D- or 3D-acquisition techniques. This was practically applied for the assessment of tracheomalacia in pediatric patients, showing similar results to bronchoscopy and CT.^30–32^Principally, dynamic 3D-imaging is favorable over 2D-imaging, since the complex movements of the trachea make it difficult to find a single slice position which covers the trachea completely at all time points throughout the respiratory cycle.^33^ Given a reasonably regular respiratory cycle, dynamic, gated reconstructions from free-breathing long-term acquisitions combine better spatial resolution and a high signal-to-noise ratio, such as dynamic UTE MRI, which has been successfully applied for the assessment of tracheomalacia in neonates without sedation.

**Figure 4.**
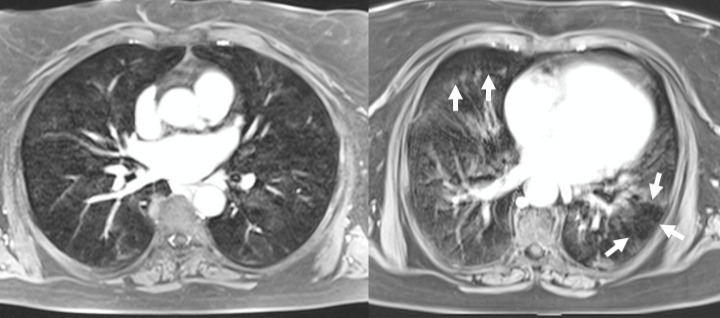
66-year-old female with COPD, air trapping in expiration. 3D-gradient echo MR scans in inspiratory (left) and expiratory breath-hold (right) obtained after iv. contrast application. The image in expiration demonstrates inhomogeneous lung signal with areas of remaining inflation and low signal as a result from air trapping (arrows).

Beyond direct visualization of large airway collapse, expiratory MRI scans can show indirect signs of small airway dysfunction. Expiratory collapse or occlusion of smaller airways with mucus results in incomplete deflation of the dependent lung tissue in expiration and produces a mosaic pattern or air trapping on expiratory scans as indirect signs of bronchiolar involvement^24^ (Figure 4). Any morphologic MRI protocol for imaging of airway-diseases should therefore contain at least one series in expiration. However, for CT, it has been reported that various degrees of air trapping can be observed in subjects with normal pulmonary function, irrespective of current smoking status and cigarette consumption.^34^ In particular in asymptomatic healthy subjects with normal pulmonary function, air trapping is usually limited to fewer than three adjacent secondary pulmonary lobules (“lobular air-trapping”).^35^ Since it has been shown that the sensitivity of MRI is lower compared to CT, it may be speculated, that such limited, clinically insignificant air trapping will not be seen with MRI, while any air trapping large enough to detect with MRI is probably clinically significant, which would make MRI the less sensitive, but potentially more specific test.^36^ Further research to prove this hypothesis might be useful.

## Small airways disease: direct imaging of airway dysfunction

The abovementioned difficulties in the assessment of small airways disease with standard pulmonary function tests becomes relevant in smoker’s COPD, for example. Current pathogenetic concepts discuss accumulation of inhaled fine airborne particles at the level of small bronchioles, where tidal gas flow decreases and diffusion becomes the main mechanism of gas exchange. Here, the persistent deposition of toxic material stimulates a chronic inflammatory immune cell infiltration. Tissue repair and remodeling start to increase airway resistance and finally narrow and reduce the terminal bronchioles leading to a relatively late decline in FEV1.^37^ Due to their small size, the direct visualization of small airway involvement is challenging for both CT and MRI. As mentioned above, mucus plugging and peri-bronchial infiltration (tree-in-bud sign) or air trapping may help, as well as the observation of air trapping on expiratory scans.^19^ *Per se*, local ventilation deficits or air trapping resulting from small airway dysfunction can be quantified with lung deformation mapping from MRI in inspiration and in expiration. This has been used to study local lung mechanics in early fibrosis, but would be principally suitable to study lung ventilation as well^38^ – although, dedicated studies or clinical evidence have not been published so far.

**Figure 5.**
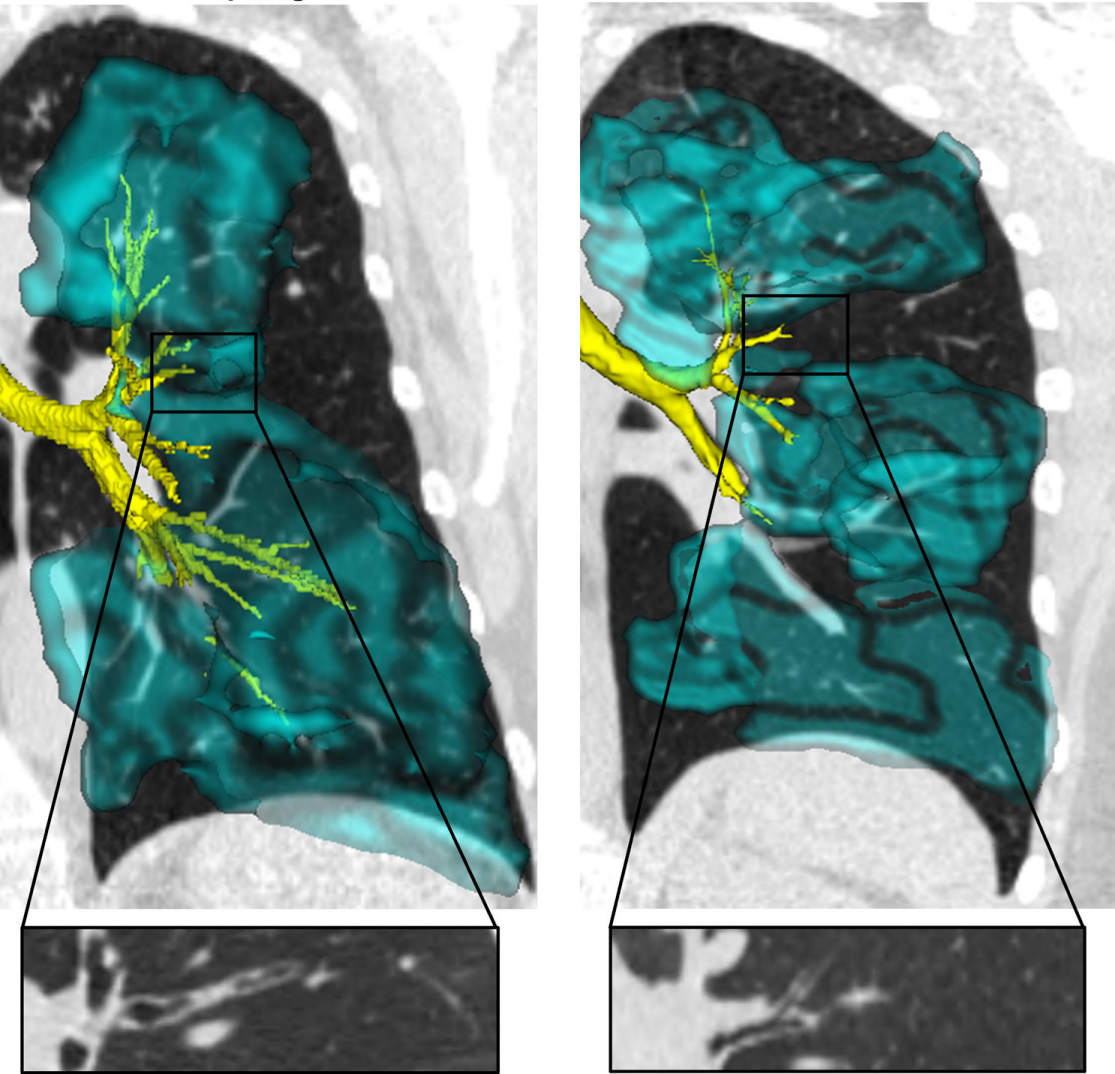
MRI ventilation deficits and relationship to airway abnormalities in two patients with severe asthma. ^129^Xe ventilation (cyan) in three-dimensions co-registered with coronal MDCT reconstructions and airway tree renderings from CT (yellow). The left panels show a left upper lobe posterior ventilation abnormality that was spatially related to an occluded airway shown in the inset with suspected mucus plugging. Right panels show a left lower lobe ventilation abnormality corresponding to airway narrowing (shown in the inset) and numerous ventilation defects distributed throughout the left lung related to airway remodelling and obliteration. Images courtesy of Marrissa J. McIntosh, Harkiran K. Kooner and Grace Parraga, Robarts Research Institute and Department of Medical Biophysics, Western University, London, Canada

In the scientific setting, direct visualization of the ventilation deficits or ventilation heterogeneity from large and small airways disease can be achieved with MRI and inhaled hyperpolarized gases, i.e.,^3^He and ^129^Xe. In Asthma, for example, the distribution of the inhaled gases correlates with airway inflammation and re-modelling, luminal occlusions or airway smooth muscle dysfunction (Figure 5).^39^In pediatric radiology, MRI with hyperpolarized gases has been used for several lung diseases, including asthma, cystic fibrosis, congenital diaphragmatic hernia, and bronchopulmonary dysplasia.^40^

Beyond visualization of the ventilated air spaces and ventilation defects from small airway dysfunction, hyperpolarized ^3^He and ^129^Xe allow for quantitative measurement of terminal airway and alveolar morphology. This is based on the fact that, *e.g*. for ^3^He near the dilute limit in air, the free (unrestricted) diffusivity is 0.88 cm^2^/s with a free displacement of 0.59 mm at a typical diffusion time of 2 ms.The diffusion of the gas is therefore restricted inside acinar ducts and acini with diameters around 0.7 mm and 0.3 mm, respectively. This effect can be assessed with appropriate MR sequences and quantified as a reduction of the apparent diffusion coefficient (ADC), compared to ^3^He outside the lung.^41^ Consequently, any expansion of the alveoli and tissue destruction associated with emphysema result in a higher ADC which correlates to the average terminal airway diameters.^42^ In COPD for instance, increased ADC in enlarged, emphysematous spaces allows for the calculation of the emphysema index.^43^ In idiopathic pulmonary fibrosis, the fibrotic changes lead to a loss of alveolar surface area, the formation of honeycomb cysts, and dilation of terminal bronchi. These conditions result in higher ^3^He diffusion and an elevation of the calculated ADC.^44^ Accordingly, a higher ADC is found in areas of air trapping and local hyperinflation of asthmatic patients’ lung.^45^ Thus, at the very last stretch of the airway tree, MRI with hyperpolarized gases even allows for quantitative assessment of terminal airways and the alveolar space dimensions.

Finally, hyperpolarized ^129^Xe also allows to assess the gas transfer into the alveolar–capillary tissue space and from the tissue barrier into red blood cells in the pulmonary microvasculature. However, the limited availability and high cost of the gases and the need of dedicated hardware and complex image analysishave so far limited MRI with hyperpolarized gases to research purposes and a broad clinical implementation is not expected in the near future.

A less complex approach uses direct imaging of the non-hyperpolarized gases with MR at the resonant frequency. The most promising element is ^19^F (inert fluorine, *e.g*. to be inhaled as a mixture of pure oxygen and SF_6_ gas) and the feasibility of this approach has been shown.^46^ The scientific value of inert ^19^F imaging has been demonstrated with a study on lung ventilation deficits in COPD patients.^47^ However, this approach does not need preparation of the gases with hyperpolarization, but it still requires appropriate MR hardware tuned to the resonance frequency of the gas. The achievable signal intensity is low due to the limited density of fluorine in the gas and the necessary robustness and evidence to adopt this promising technique for clinical imaging still need to be proven.

Alternatively, the T1-shortening effect of oxygen can be used to assess lung ventilation. Basically, this needs only a state-of-the-art MR scanner and medical oxygen, which should be easily available in any hospital. For the assessment of lung ventilation, two series of multiple images while breathing room air or oxygenated air are acquired, respectively. The signal intensity on the resulting subtracted images correlates with the elevated oxygen concentration inside the ventilated alveolar space. Oxygen-enhanced MRI has already been used to assess lung ventilation in adults and children, but poorer SNR and the long acquisition time of this technique have so far hampered its broader use in the clinic.^48,49^ Significant improvements are expected from recent work at lower field strength with potentially higher signal intensities in the lung tissue, which might draw more attention on this very useful approach.^50^

Finally, it has been hypothesized that even plain, non-contrast-enhanced MRI might be suitable to quantify blood content and aeration separately, since both contribute to the MR signal characteristics of the lung parenchyma. In fact, T1* measurements in patients in COPD and asthma have shown encouraging results.^51,52^ These approaches are highly experimental and based not only on the measurement of T1 itself but also on effects such as T1-dependence on the applied echo time TE. An adoption for clinical imaging in the near future is not expected.

**Figure 6.**
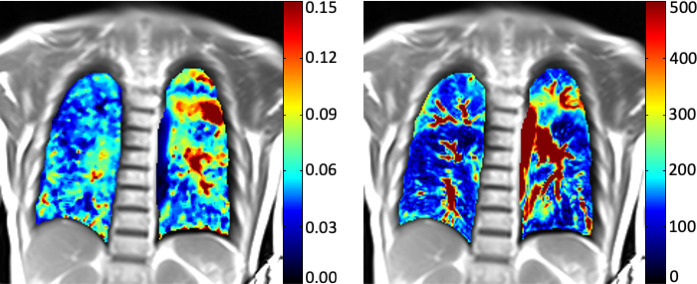
Ventilation-perfusion MRI using Fourier-decomposition (Matrix Pencil MRI/MP-MRI) in a 60-year-old COPD patient (GOLD III/IV), fractional ventilation map (left) and perfusion map (right, perfusion in ml/100 ml/min.). In correlation with extensive parenchymal destruction, the color-encoded maps show marked inhomogeneities of ventilation and perfusion over large parts of the lung. *Images courtesy of Grzegorz Bauman, Division of Radiological Physis, Department of Radiology, University of Basel Hospital, Switzlerland*

Other, non-contrast-enhanced proton MRI techniques exploit the periodic signal changes of the lung resulting from respiration and blood flow pulsation. Fourier decomposition MRI, for example, is based on 2D or 3D gradient echo non-contrast-enhanced chest MRI acquisitions technique with high temporal resolution (3–4 images per second) obtained in free breathing without cardiac or respiratory gating.^53,54^ To eliminate respiratory motion, the images are registered to the inspiratory or expiratory frame or at any favorable position in between. Then, signal intensity changes of the lung parenchyma related to the cardiac and respiratory cycle are decomposed using a Fourier transform to obtain perfusion- and ventilation-weighted images. Besides Fourier MRI, other approaches with comparable diagnostic scope have been developed (*e.g.,* SENCEFUL, PREFUL or matrix pencil MRI).^53,55,56^ Such completely non-invasive techniques are particularly useful for scientific work with healthy subjects, for example to assess small airways disease in smokers after exposure to tobacco fume or vaping electronic cigarettes without application of intravenous orgaseous contrast agents (Figure 6).^55^ Fourier decomposition imaging has also been tested in children with CF and asthma and obtained similar diagnostic information to hyperpolarized gas MRI or dynamic contrast-enhanced MRI.^49^ All these dynamic imaging techniques allow for imaging both, ventilation and lung perfusion in one study, which makes them the most comprehensive, non-contrast enhanced low-cost imaging approach and very promising candidates to replace contrast-enhance functional imaging of the lungs in the near future.

## Small airways disease: indirect imaging of airway dysfunction

Indirect imaging of airway dysfunction is based on the physiological process of hypoxic vasoconstriction in the human lung. This mechanism triggers constriction of intrapulmonary arteries in response to alveolar hypoxia, diverting blood to better-oxygenated lung segments, thereby optimizing ventilation/perfusion matching and systemic oxygen delivery.^57^ This means that any kind of airway obstruction produces a corresponding perfusion deficit in the dependent lung. Therefore, any technique for the assessment of lung perfusion can be used to indirectly assess airway dysfunction by the detection of the resulting perfusion deficits.

Compared to other techniques, dynamic first-pass perfusion MRI (DCE MRI) is easy to implement and provides a reasonably robust image quality for this purpose. It produces a time-resolved image series of pulmonary contrast transit using an intravenously injected contrast material bolus and multiple acquisitions of lung volumes with a very fast 3D gradient echo sequence.^58^ Reasonably short acquisition times of 1.5 s or less per full lung volume are achieved by combining parallel imaging and data sharing.^8^ The resulting images can be evaluated with a dynamic display. For quick review, subtraction of the baseline images from the contrast-enhanced series facilitates the detection of perfusion deficits. Airway dysfunction is indicated by perfusion deficits related to hypoxemic vasoconstriction in areas of reduced or missing pulmonary ventilation.^59,60^ However, the images need careful interpretation, since before assuming perfusion deficits from hypo-ventilation, lung parenchyma defects (*e.g.,* from emphysema), or vessel occlusion from pulmonary embolism need to be excluded. Acquisition of morphologic and angiographic image series in the same study and review together with the DCE series are therefore mandatory. This is, for example, needed in COPD patients, where perfusion deficits from emphysema and small airways disease overlap.^61^

The fact that DCE MRI provides information on both, perfusion and ventilation deficits of the lung, that have made is the so far most widespread functional imaging approach and a common component of clinical lung MRI protocols.^25,40^ On the other hand, the intravenous application of gadolinium-based MR contrast material is controversial and alternative techniques would be appreciated.^62^ DCE MRI has been implemented, for example, to monitor lung ventilation and perfusion deficits after physiotherapy and antibiotic medication of children with cystic fibrosis (CF, Figure 2).^21^ Fully automated quantification software of the perfusion studies to convert this image information into useful clinical scores (image-based biomarkers) is in development, but not yet commercially available.^61^ In the meantime, semi-quantitative visual scoring systems have already found broad clinical use.^26^

However, any technique for the assessment of lung perfusion can be used for the indirect approach. For instance, arterial spin labeling (ASL) is based on the signal from tagged blood inflow into a pre-saturated, signal-inverted volume and produces perfusion maps that indirectly reflect the effects of hypoxic vasoconstriction.^63^ This has been successfully applied for physiologic studies in healthy subjects, for example to assess the effects of hypoxia and to investigate the effects of hypoxic vasoconstriction on pulmonary blood flow heterogeneity.^64^ A broad clinical use of ASL in the lungs has so far not been reported.

## Airway imaging with common clinical MRI protocols

The currently available MRI protocols from standard clinical equipment already cover many aspects of airway imaging. Detailed protocol descriptions can be found in the literature with adaptions for use in adult^8^ and pediatric patients.^9^ Briefly, common standard protocol suggestions include *T*_2_-weighted fast spin echo (FSE) sequences, steady-state free precession (SSFP) sequences for respiratory motion and *T*_1_-weighted 3D gradient echo (3D-GRE) sequences in breath-hold. Adjusted to the size of the patient, the fields of view (FOVs) are typically 450–500 mm in coronal and approximately 400 mm in transverse acquisitions with matrices of 256–384 pixels (for triggered FSE up to 512) and pixel sizes smaller than 1.8 × 1.8 mm. Suitable slice thicknesses are 4 to 6 mm for the 2D acquisitions, 4 mm or less for 3D acquisitions in transverse and 2 mm or less in coronal orientation (as would be applied for pulmonary angiography).^8^ This provides a sufficient spatial resolution for the *assessment of the tracheobronchial morphology*, as introduced above. Acquisition in inspiratory and expiratory breath-hold facilitates the detection of airway collapse and air trapping.

Another standard component is the aforementioned DCE MRI with fast, time-resolved 3D gradient echo (3D-GRE) acquisitions. Standard protocol suggestions recommend to use a combination of the time-resolved, low spatial resolution acquisition for first pass perfusion imaging with a high spatial resolution acquisition for a breath-hold angiogram.^8^ The dynamic study produces a comprehensive lung perfusion study with excellent temporal resolution and at the same time serves for the determination of optimum contrast bolus timing for the acquisition of the high-resolution angiogram.

This is used for the *indirect visualization of ventilation disorders* appearing as lung perfusion deficits from hypoxic vasoconstriction. As discussed, this requires exclusion of lung parenchyma defects and pulmonary embolism. Therefore, it is recommended to acquire and interpret DCE MRI only in combination with morphologic sequences and a standard MR angiography, as described above.

These protocols are either already pre-installed on the systems or can be quickly assembled from standard sequence packages for abdominal and cardiac MRI. For the near future, it can be expected that the more complex procedures outlined in this article (*e.g.,* imaging with hyperpolarized gases) will become step by step available for clinical use as well.

## Conclusions

Lung MRI is finding increased world-wide acceptance as part of clinical routine and for research imaging in cystic fibrosis, asthma, COPD and other airway diseases. Airways are directly visualized down to the fourth order while bronchiectasis, wall thickening, and mucous plugging increase the visibility of even much smaller airways. Mosaic perfusion and air trapping are indirect signs of small airways disease. Ventilation inhomogeneities resulting from small airways disease can be indirectly visualized as lung perfusion deficits after iv.-contrast (reflecting hypoxic vasoconstriction) or directly with hyperpolarized noble gases, inert fluorine or oxygen-enhanced imaging. Non-contrast enhanced ventilation-perfusion imaging techniques complete the bunch of promising approaches, each of them with different levels of development and clinical feasibility. At the very last stretch of the airway tree, for research purposes, MRI with hyperpolarized gases even allows for measurements of terminal airways and the alveolar space.This makes MRI the most comprehensive imaging modality covering all aspects of airway morphology and function and allows to use it for scientific purposes and an increasing number of clinical indications as well- with semi-quantitative visual scores or automated quantitative evaluation producing useful biomarkers for clinical decisions.
